# Characterizing Lung Disease in Cystic Fibrosis with Magnetic Resonance Imaging and Airway Physiology

**DOI:** 10.1371/journal.pone.0157177

**Published:** 2016-06-23

**Authors:** Rebecca J. Theilmann, Chantal Darquenne, Ann R. Elliott, Barbara A. Bailey, Douglas J. Conrad

**Affiliations:** 1 Department of Radiology, University of California San Diego, San Diego, California, United States of America; 2 Department of Medicine, University of California San Diego, San Diego, California, United States of America; 3 Department of Mathematics and Statistics, San Diego State University, San Diego, California, United States of America; University of Alabama at Birmingham, UNITED STATES

## Abstract

Translational investigations in cystic fibrosis (CF) have a need for improved quantitative and longitudinal measures of disease status. To establish a non-invasive quantitative MRI technique to monitor lung health in patients with CF and correlate MR metrics with airway physiology as measured by multiple breath washout (MBW). Data were collected in 12 CF patients and 12 healthy controls. Regional (central and peripheral lung) measures of fractional lung water density (FLD: air to 100% fluid) were acquired both at FRC and TLC on a 1.5T MRI. The median FLD (mFLD) and the FRC-to-TLC mFLD ratio were calculated for each region at both lung volumes. Spirometry and MBW data were also acquired for each subject. Ventilation inhomogeneities were quantified by the lung clearance index (LCI) and by indices S_cond_* and S_acin_* that assess inhomogeneities in the conducting (central) and acinar (peripheral) lung regions, respectively. MBW indices and mFLD at TLC (both regions) were significantly elevated in CF (p<0.01) compared to controls. The mFLD at TLC (central: R = 0.82) and the FRC-to-TLC mFLD ratio (peripheral: R = -0.77) were strongly correlated with S_cond_* and LCI. CF patients had high lung water content at TLC when compared to controls. This is likely due to the presence of retained airway secretions and airway wall edema (more water) and to limited expansions of air trapping areas (less air) in CF subjects. FRC-to-TLC ratios of mFLD strongly correlated with central ventilation inhomogeneities. These combined measures may provide a useful marker of both retained mucus and air trapping in CF lungs.

## Introduction

Cystic fibrosis (CF) is an inherited disease affecting over 30,000 individuals in North America and is associated with a median age of death at ~41 years [1]. CF is caused by mutations in the CFTR gene, resulting in ineffective deployment and function of the mucus layer overlying airway epithelium leading to impaired mucociliary clearance [2]. Pulmonary function studies and in particular spirometry, have been used for decades to follow disease progression. Yet, CF is a disease that originates in the small airways, a region of the lung that is difficult to assess with spirometry [3]. Thus, there is a critical need for new approaches to better capture abnormalities in the small airways to identify the progression of early disease and smaller but clinically important changes in lung physiology.

CF is characterized by the accumulation of viscous purulent airway secretions that leads to airway obstruction, chronic infections, and airway inflammation [4–6]. Each of these traits may have the potential to alter the regional lung water content when compared with controls. Though dehydrated when compared to normal subjects (~90% water), the mucus layer that obstructs CF airways is approximately 80% water. Due to the failure to clear mucus from airway surfaces, airway surface plaques are produced that accumulate with continued mucin secretions from goblet cells and/or glands [7, 8]. With time, excess airway secretions would be expected to increase the regional lung water content. Based on previous studies [9, 10], airway inflammation may also increase the regional lung water content. Bronchiectasis is defined as a large airway filled with secretions containing mucus which are 85% water and 15% solid [11]. MRI, with its intrinsic sensitivity to the regional water content, provides a promising approach to visualize these characteristics of CF, especially since there are no concerns regarding ionizing radiation as with PET and CT. Proton MRI detects the presence of hydrogen atoms (protons), and since many of these protons are protons in water, the relative signal intensity is directly proportional to the local water content. In healthy subjects, lung water content is a measure of water from blood and lung tissue; in CF, it also includes water from excess airway secretions and lung abnormalities. Thus, quantifying lung water content may be potentially useful in the monitoring of the disease.

It is well know that CF patients have abnormal lung function due to abnormal airway morphology. The use of multiple breath washout (MBW) to assess lung function has recently regained the interest of the CF research community mainly because its derived lung clearance index (LCI) has been considered a marker of small airway function. Other MBW indices, S_cond_ and S_acin_, have been used in various lung diseases [12–16] to assess ventilation inhomogeneities in the conducting (central) and acinar (peripheral) regions of the lung, respectively. These indices, while not commonly used in the clinic, may provide a better characterization of regional ventilation inhomogeneities.

We hypothesize that excess water content quantified by MRI in the adult CF lung with stable disease is a marker of abnormal lung morphology and that this metric is linked with lung function as measured by MBW.

## Materials and Methods

### Study Population

A total of 12 CF subjects with stable disease were recruited from the University of California, San Diego (UCSD) Adult CF clinic by the clinical director (D.J.C.). The subjects had no significant changes in their symptoms for the 4 weeks prior to the study and had no recent change in their therapy. Twelve healthy adults without CF were recruited from the local population in San Diego. All subjects provided written informed consent approved by the Human Research Protections Program at the University of California, San Diego (#110368).

### Spirometry

All subjects performed spirometry following American Thoracic Society standards (Easy One spirometer, Zurich, Switzerland). Predicted values were based on the Third National Health and Nutrition Examination Survey reference values [17].

### Multiple Breath Nitrogen Washout (MBW)

The MBW test was performed on a dedicated assembly similar to that previously described [12, 14] and followed the ERS/ATS recommendations in terms of equipment and analysis of outcome variables [18]. The equipment was originally developed by Verbanck and colleagues and is currently used in several laboratories worldwide [12, 19, 20]. Briefly, the system uses a bag-in-box system with separate bags for inspired and expired gases. The subject breathes through a non-rebreathing valve, inspiring air or test gas (100% O_2_). Flow and gas concentrations are measured with a pneumotachograph located in the wall of the bag-in-box system and a rapid-responding mass spectrometer located near the lips of the subject, respectively. The system was calibrated before and after each session.

The test required a regular breathing pattern using 100% O_2_ for inspiration while respired volume and N_2_ concentration were continuously recorded at the mouth. The test required approximately 25–30 breaths until the mean expired N_2_ concentration fell to 2% so that the lung clearance index (LCI) could be calculated. LCI is a measure of gas mixing efficiency calculated from the relative ventilation required to clear a tracer gas (in this case N_2_) from the lung and is defined as the number of lung turnovers (ie, number of FRC) required to reach 1/40th of the starting concentration of the tracer gas. Tests were performed in triplicate.

In addition to LCI, S_cond_, and S_acin_ were calculated to assess ventilation inhomogeneities in the conducting (central) and the acinar (peripheral) regions of the lung. These indices increase when ventilation heterogeneity increases. In particular, in healthy subjects, S_acin_ reflects ventilation inhomogeneity resulting from a normal peripheral lung structure with a given degree of asymmetry while S_cond_ results from a given difference in ventilation between lung units subtended from conductive airways and/or if there is a increased degree of unequal emptying of these units. Whenever S_acin_ undergoes significant changes with respect to baseline, this is due to an important alteration in the peripheral lung structure [15], such as unequal lumen narrowing of inflamed respiratory bronchioles. Whenever S_cond_ is increased, there has been a change in the conductive airways or the pressure-volume characteristics of the lung units subtended by these conductive airways.

LCI and alveolar slope of N_2_ concentration for each expiration was calculated using automated software [20] using standard methodology [21–23]. Because of the high level of ventilation heterogeneities present in the lungs of CF patients, we used the method described by Verbanck and colleagues to calculate a modified S_acin_* and S_cond_* [24]. An average of each metric were presented for data calculated with a R^2^ > 0.5 in the logistic regression for S_cond_ between the chosen turnovers (i.e 1.5 and 3).

### Multiple Gradient Echo Magnetic Resonance Imaging (mGRE MRI)

Imaging was carried out on a 1.5 Tesla GE HDx EXCITE twinspeed scanner (Milwaukee, WI) using a validated multi-gradient echo (mGRE) sequence previously described by Theilmann et al [25, 26]. To quantify measures of lung water, the mGRE imaging sequence acquires multiple single echo acquisitions alternating between two echo times in a single 9-second breath-hold. A reference phantom has been placed on the anterior chest wall so as to be within the field of view of the scans, permitting absolute quantification of lung water density. Lung water density is determined by back extrapolating the signal to an echo time of zero (e.g. TE = 0) by fitting data to a single exponential. Since the phantom does not have decay constants equal to that in the lung, a correction factor is empirically determined for the static sequence parameters (T_R_ = 10ms, T_2_ decay effects negligible) permitting the mean phantom signal to be used as a reference for absolute calibration of water content. Imaging sequence parameters are T_R_ = 10 msec, flip angle = 10 deg, slice thickness = 15 mm, field of view = 40 cm, receiver bandwidth = 125 kHz, first echo time (TE_1_) = 1.0 msec, second echo time (TE_3_) = 1.8 msec, and a full acquisition matrix of 64 x 64. All imaging parameters were kept within the established FDA guidelines for routine clinical MR examinations.

**Fig 1:**
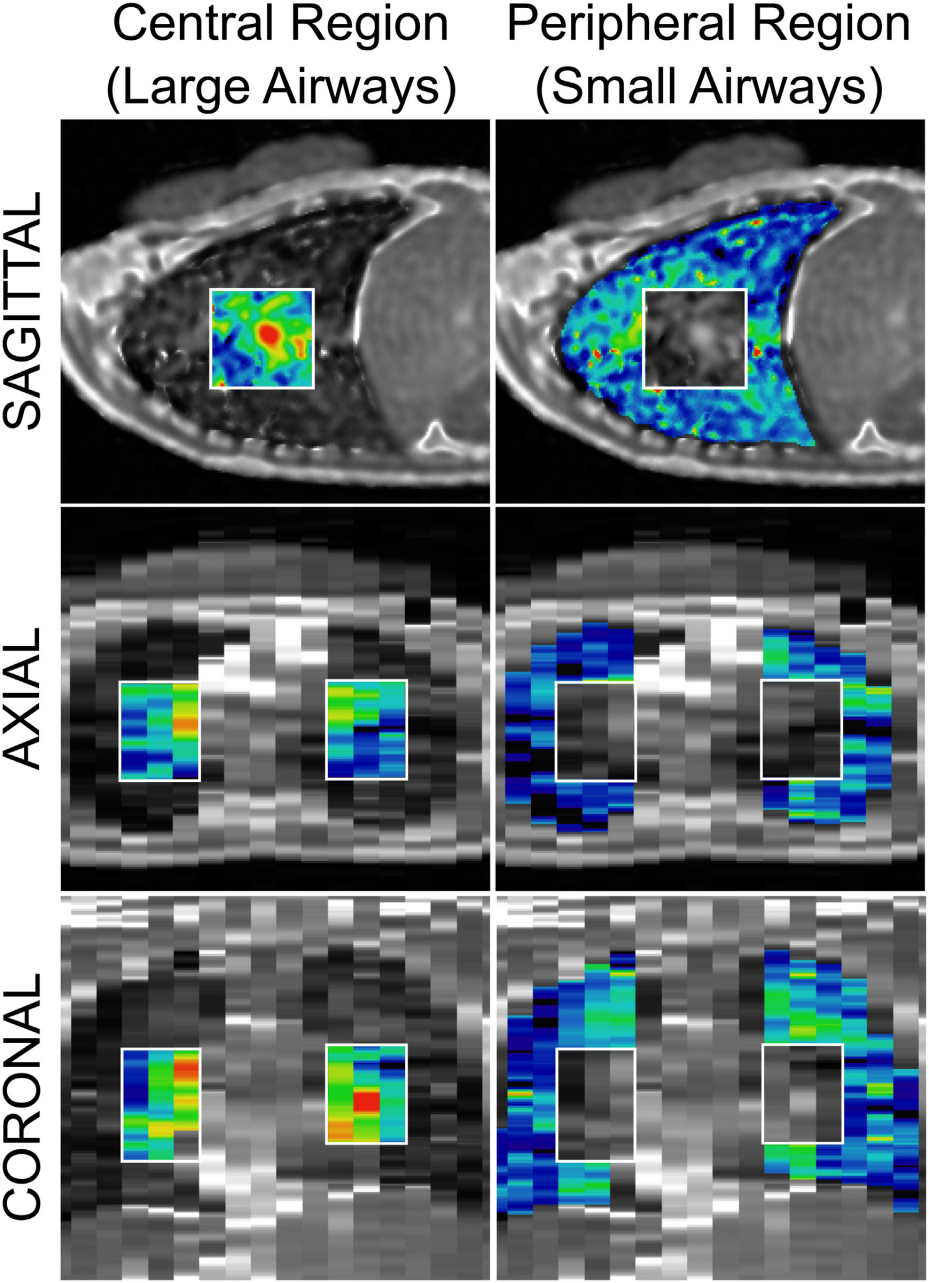
Representative fractional lung density maps at total lung capacity (TLC) of a control subject and three CF subjects with stable disease. Sagittal FLD maps of the entire lung at TLC are shown for a healthy control (F, 42 yo, FEV_1_ = 104%), a CF subject with mild disease (F, 30yo, FEV_1_ = 86%) a CF subject with moderate disease (F, 27yo, FEV_1_ = 53%), and a CF subject with severe disease (M, 24yo, FEV_1_ = 32%). As expected for the control subject, lung water density is relatively uniform throughout the lung at TLC with an FLD greater than 0.15 indicating large blood vessels. When compared to the healthy subject, locations of increased water content become discernible for each of the CF subjects with the spatial extent and the amount of water increasing with disease severity. Note: color overlay represents spatial location in the lung included in the analysis.

Three 2D sagittal slices of image data were acquired within successive 9 sec breath holds. Each subject completed 20 (or 24) individual breath holds to obtain image data of the lung in duplicate both at functional residual capacity (FRC) and total lung capacity (TLC). All imaging was completed within a 30–40 minute exam. Image data was converted to a quantitative measure of fractional lung water density (FLD) using the signal of the reference phantom as a measure of signal without any partial volume effects (i.e. 100% water). FLD was calculated on a voxel-by-voxel basis and as a fraction of water ranging from 0 (air) to 1 (100% fluid). Representative FLD maps of a control subject, and three CF subjects (mild, moderate, and severe disease) at TLC are shown in Fig 1.

**Fig 2:**
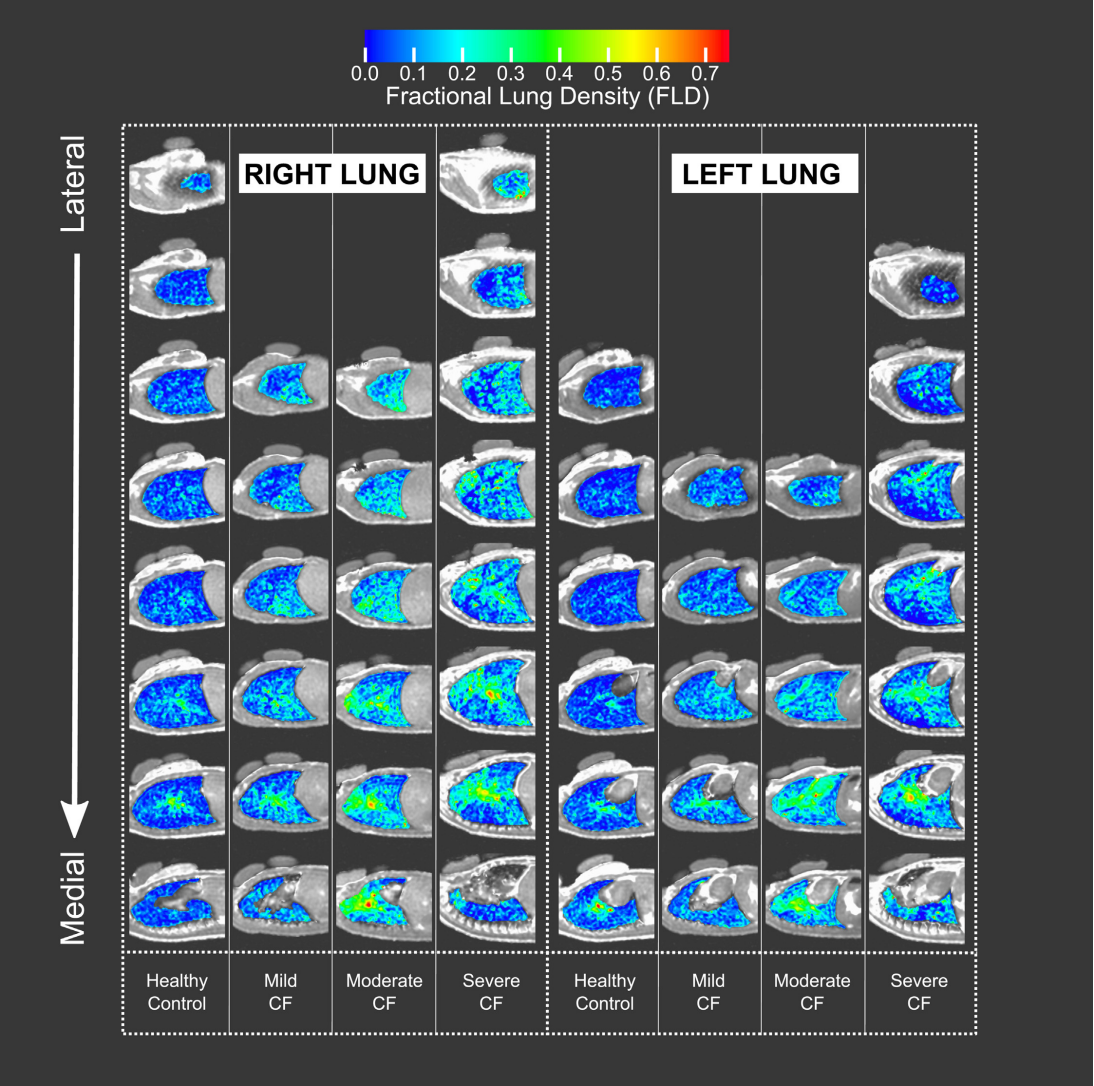
Diagram of central and peripheral lung regions. The central and peripheral regions (identified in color within white outline) are defined in the same manner as in [27]. For each lung, two outline region of interests (ROIs) are created over the lung image: 1) a rectangular ROI around the entire lung and 2) a central ROI, with dimensions equal to half the whole lung ROI’s width and one-half its height. The central region is positioned on the interior boundary of the lung, with the peripheral region being the area lying between the central and whole lung outline.

Regional FLD in the central and peripheral region of the lung were assessed for data obtained at both FRC and TLC. The central and peripheral regions (Fig 2) were mathematically defined as previously published [27]. The median of the FLD distribution (mFLD) is reported for each region (central, peripheral), each repetition, and each lung volume (FRC, TLC).

### Statistical Analysis

Statistical analysis was performed using R package ‘stats’ (version 3.1.0). Analysis of variance for repeated measures was used to statistically evaluate change in the major dependent MRI variable (mFLD) over 2 repeated measures: (i) lung volume (2 levels: FRC and TLC), (ii) spatial location within lung (2 levels: central and peripheral region), and (iii) subjects group (2 levels: controls and CF). Where overall significance occurred, post-hoc testing was conducted using Student’s t-tests. Student’s t-tests were used to statistically evaluate the major dependent MBW variables (S_cond_*, S_acin_*, and LCI) between CF and controls. All data are presented as means, SD, and the null-hypothesis (no effect) was rejected for P <0.05 (2-tailed), except as indicated.

Linear regression analyses were employed to explore the relationship between MBW indices (dependent variable) and MRI structural metrics (independent variables). Data are presented as correlation (R) and the significance of R for both subjects pooled together (all subjects) and also for the CF subjects only. Given the complicated morphology of the CF lung, it is highly unlikely that a single type of defect would alter measures of lung function as measured by MBW. Thus, hierarchical multiple regression analyses were implemented to explore which combination of MRI metrics in the CF lung affect MBW indices, LCI and S_cond_*.

## Results

### Subject Characteristics

Table 1 summarizes the characteristics of the CF and control group. Twelve CF patients (6M/6F) and twelve healthy adults (4M/8F) were recruited for the study. Within the CF subject population (32% < FEV_1_ < 104%), four had mild disease (FEV_1_ > 70%), five had moderate disease (50% < FEV_1_ < 70%), and three had severe disease (FEV_1_ < 50%). The control group had normal lung function data (86% < FEV_1_ < 105%). The CF group had significantly lower weight (kg), FEV1 [%pred], FVC [%pred], and FEF25-75 [%pred] when compared with the control group.

**Table 1:** Characteristics of CF Subjects and Non-CF Control Subjects.

Characteristics	Controls Subjects	CF Subjects	p-value—Subject Group
**Number of subjects**	12	12	
**Age, yr**	32.5 (8.2)	28.3 (5.3)	0.16
**Male/female, no./no.**	6/6	4/8	
**Weight, kg**	71.5 (10.2)	62.7 (7.9)	0.03
**Height, cm**	172.1 (7.6)	169.9 (8.5)	0.52
**BMI, kg/m^2^**	26.6 (9.9)	21.5 (1.8)	0.10
**FEV_1_, %predicted**	93.3 (6.2)	69.6 (23.9)	0.006
**FVC, %predicted**	94.4 (7.2)	80.1 (18.8)	0.03
**FEF_25-75%_, %predicted**	90.0 (18.8)	53.5 (37.4)	0.008

Values presented as mean(±SD). Definition of abbreviations: BMI = body mass index, CF = cystic fibrosis

Study participants performed a MRI examination, a MBW test, and spirometry at a single visit. All subjects performed all investigations except one severe CF subject (FEV_1_[%pred] = 33%) on oxygen therapy who was only able to complete one repetition out of two MRI examination.

### MBW and MRI Data

All MBW indices (LCI, S_cond_*, and S_acin_*) were significantly larger in CF than in controls (Table 2). A Pearson correlation indicated a significant test-retest reliability for the LCI (R = 0.94, p<0.0001), S_cond_* (R = 0.80, p<0.0001), and S_acin_* (R = 0.97, p<0.0001) across all subjects (n = 24).

**Table 2:** Multiple Breath Washout (MBW) Indices of CF Subjects and Non-CF Control Subjects.

	Controls		Cystic Fibrosis		
	Mean(±SD)	Range	Mean(±SD)	Range	p-value
**LCI**	6.0 (0.3)	5.5–6.8	9.9 (3.9)	6.0–18.0	0.005
S_cond_* (1/L)	0.03 (0.01)	0.01–0.05	0.14 (0.06)	0.05–0.21	0.00003
S_acin_* (1/L)	0.11 (0.04)	0.05–0.20	0.31 (0.28)	0.09–0.96	0.04

The pulmonary thoracic space [28] which is the physical volume that the lung occupies in the thoracic cavity (lung gas and tissue volumes), was determined via manual segmentation of MR images, where regions of interest (ROIs) were manually inscribed within the thoracic cavity while excluding the heart, diaphragm, and liver. Subjects with CF had a significantly lower lung volume (e.g. gas volume) at FRC as measured by MBW when compared with controls (2.4±0.6 vs. 3.2±0.7, p<0.009) but had a significantly higher total lung volume at FRC as measured by MRI (2.21±0.68 vs. 1.43±0.48, p<0.03). The total lung volume at TLC as measured by MRI was not significantly different between groups (3.77±0.80 vs. 4.00±0.63, p<0.66).

**Fig 3:**
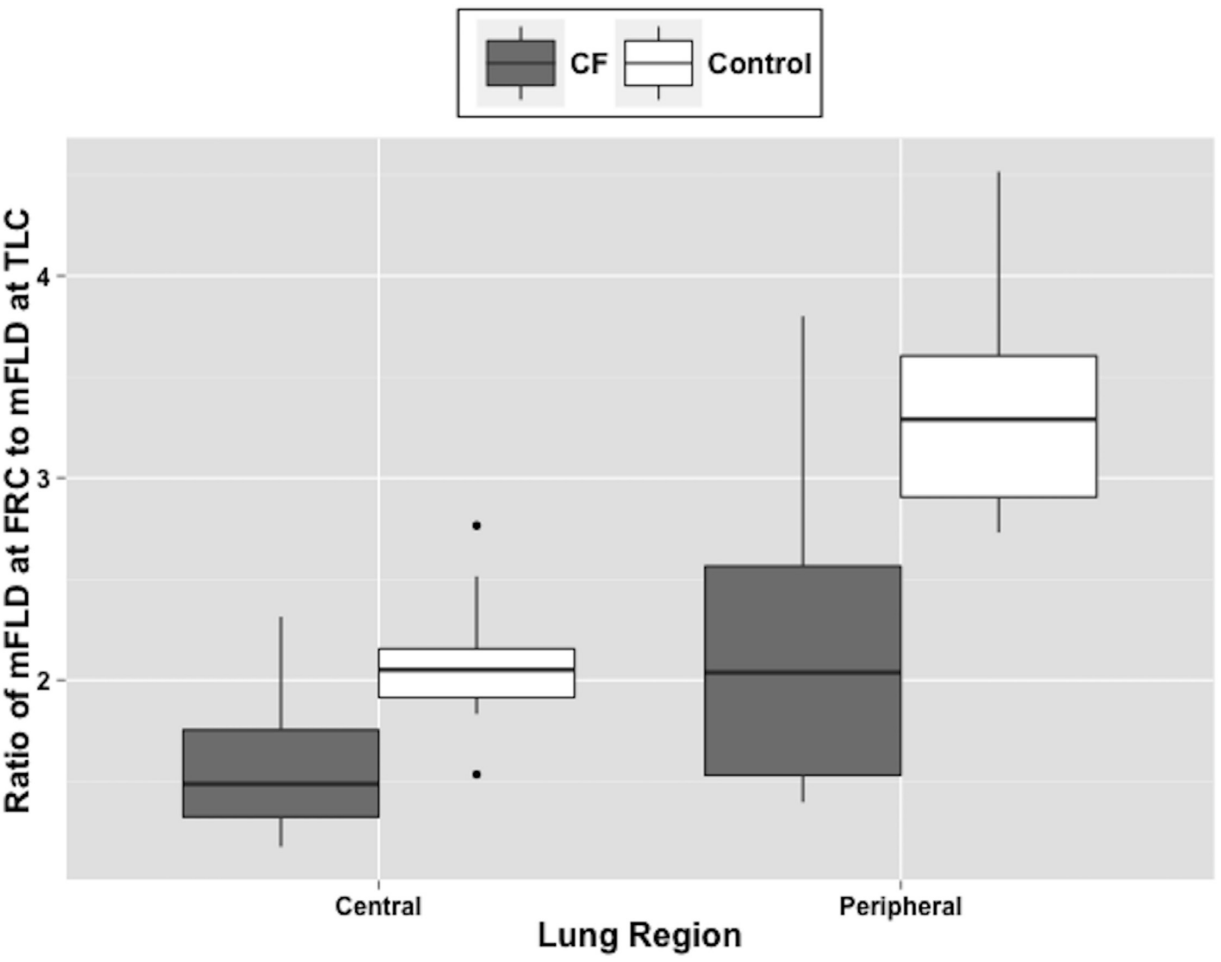
Effects of lung region on estimates of the ratio of mFLD at FRC to mFLD at TLC (mFLD ratio) for adults with CF and healthy adult controls. There was a significant main effect between groups (p<10^−24^) and between lung regions (p<10^−36^). There was also a significant lung region by group interaction (p<10^−31^).

In both groups, the median fractional lung density significantly decreased from FRC (mFLD_FRC_) to TLC (mFLD_TLC_, p<10^−30^, Table 3). Also, mFLD was larger in the central region than in the peripheral region of the lung for both groups (p<10^−15^). These changes were significantly different at TLC but not at FRC (lung volume by lung region interaction, N.S.). There was a significant group by lung volume interaction (p<0.001). Consequently, the calculated ratio of mFLD_FRC_ to mFLD_TLC_, the mFLD ratio, was significantly reduced in CF for both the central and peripheral regions when compared with controls (p<0.0001). There was also a significant group by lung region interaction (p<10^−31^) of the mFLD ratio (Fig 3).

**Table 3:** MRI Metrics of CF Subjects and Non-CF Control Subjects.

Lung Region	MRI Metric	Controls	Cystic Fibrosis	p-value
**Central Region**	**mFLD_FRC_**	0.23 (0.03)	0.25 (0.03)	0.20
	**mFLD_TLC_**	0.11 (0.01)	0.18 (0.04)	0.0006
	**mFLD Ratio**	2.09 (0.32)	1.43 (0.24)	0.0008
**Peripheral Region**	**mFLD_FRC_**	0.19 (0.03)	0.17 (0.03)	0.42
	**mFLD_TLC_**	0.06 (0.01)	0.10 (0.03)	0.002
	**mFLD Ratio**	3.36 (0.55)	1.93 (0.54)	0.0001

A Pearson correlation indicated a significant test-retest reliability for the pulmonary thoracic space (R = 0.93, p<0.0001) calculated from MR images, mFLD for each region and lung volume (R = 0.83, p<0.0001), and the mFLD ratio (R = 0.95, p<0.0001).

### Relationship between MRI and MBW

**Fig 4:**
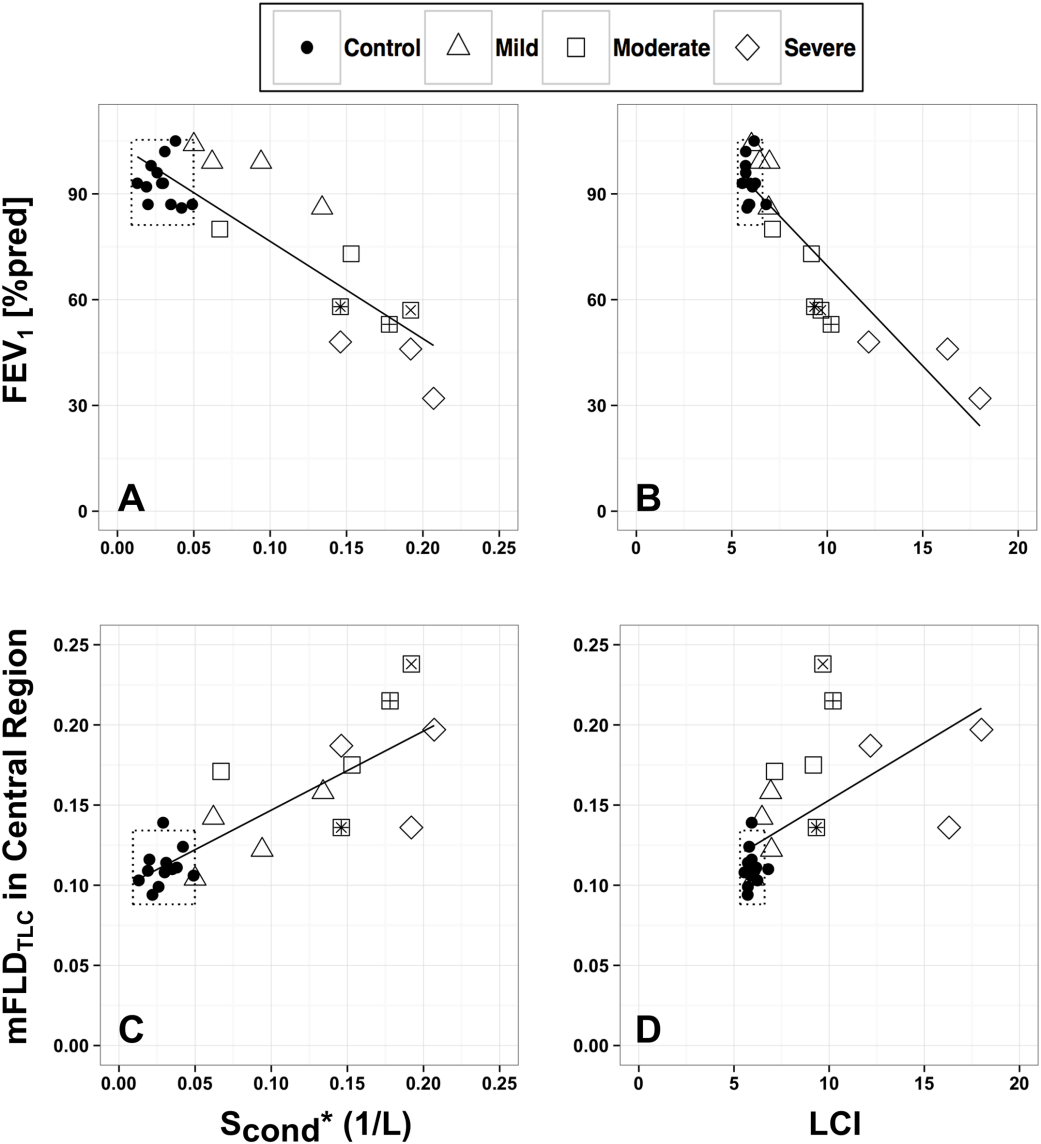
MBW indices (S_cond_*, LCI) vs. FEV_1_ [%pred] and the median fractional lung density (mFLD) in the central region (large airways) at total lung capacity (TLC). (A,C) S_cond_* (1/L) and (B,D) LCI vs. (A,B) FEV_1_ [%pred] and (B,D) mFLD_TLC_. Data presented for 12 healthy controls 12 CF adults with stable disease (4 had mild (FEV_1_ > 80%), 5 had moderate disease (50%<FEV_1_<80%), and 3 had severe disease (FEV_1_ < 50%)). Dotted lines are range of values for the control subjects in study (i.e mean ± 1.96*SD). Individual subjects with similar FEV_1_ [%pred] and LCI 2% are identified by +, * and ×.

Correlation and multiple regression analyses were conducted to examine the relationship between MBW indices (LCI, S_cond_*, and S_acin_*) and various potential predictors as measured by MRI. The predictors selected were the mFLD at each lung volume and lung region, and the mFLD ratio (Table 4). There was a high positive correlation between mFLD_TLC_ in the central lung region and S_cond_* for all subjects (R = 0.82, p<0.001) and a moderate positive correlation for CF subjects only (R = 0.64, p<0.007, Fig 4, panel C) [29]. To a lesser extent, there was a moderate positive correlation between mFLD_TLC_ in the peripheral lung region and S_cond_* for all subjects (R = 0.69, p<0.001). Compared to healthy subjects, both mFLD and S_cond_* were elevated in all CF subjects even in those who had normal FEV_1_ and LCI (Fig 4, panels B and C). Accordingly, the healthy and CF groups span a distinct range on the mFLD-S_cond_* curve.

**Table 4:** Data derived from mFLD and MBW in the central and peripheral region of the lung.

	Zero Order Correlation with^#^							
	All Subjects (n = 24)				CF Subjects Only (n = 12)			
mFLD	FEV_1_ (%pred)	S_cond_* (1/L)	S_acin_* (1/L)	LCI	FEV_1_ (%pred)	S_cond_* (1/L)	S_acin_* (1/L)	LCI
**Central Region**								
** FRC**	-0.15	0.19	-0.16	0.05	0.12	-0.13	-0.47	-0.21
** TLC**	-0.75 §	0.82 §	0.35	0.60 ‡	-0.62 †	0.64 †	0.05	0.34
** Ratio**	0.72 §	-0.79 §	-0.49 †	-0.64 §	0.83 §	-0.85 §	-0.40	-0.57
**Peripheral Region**								
** FRC**	0.34	-0.30	-0.52 †	-0.45 †	0.53	-0.49	-0.71 ‡	-0.62 †
** TLC**	-0.62 ‡	0.69 §	0.27	0.50 †	-0.46	0.43	0.00	0.22
** Ratio**	0.70 §	-0.79 §	-0.50 †	-0.67 §	0.77 ‡	-0.75 ‡	-0.38	-0.57

^#^ Pearson’s coefficient of correlation between ROI value and lung function values.

^†^ p<0.05,

^‡^ p<0.01,

^§^ p<0.001

**Fig 5:**
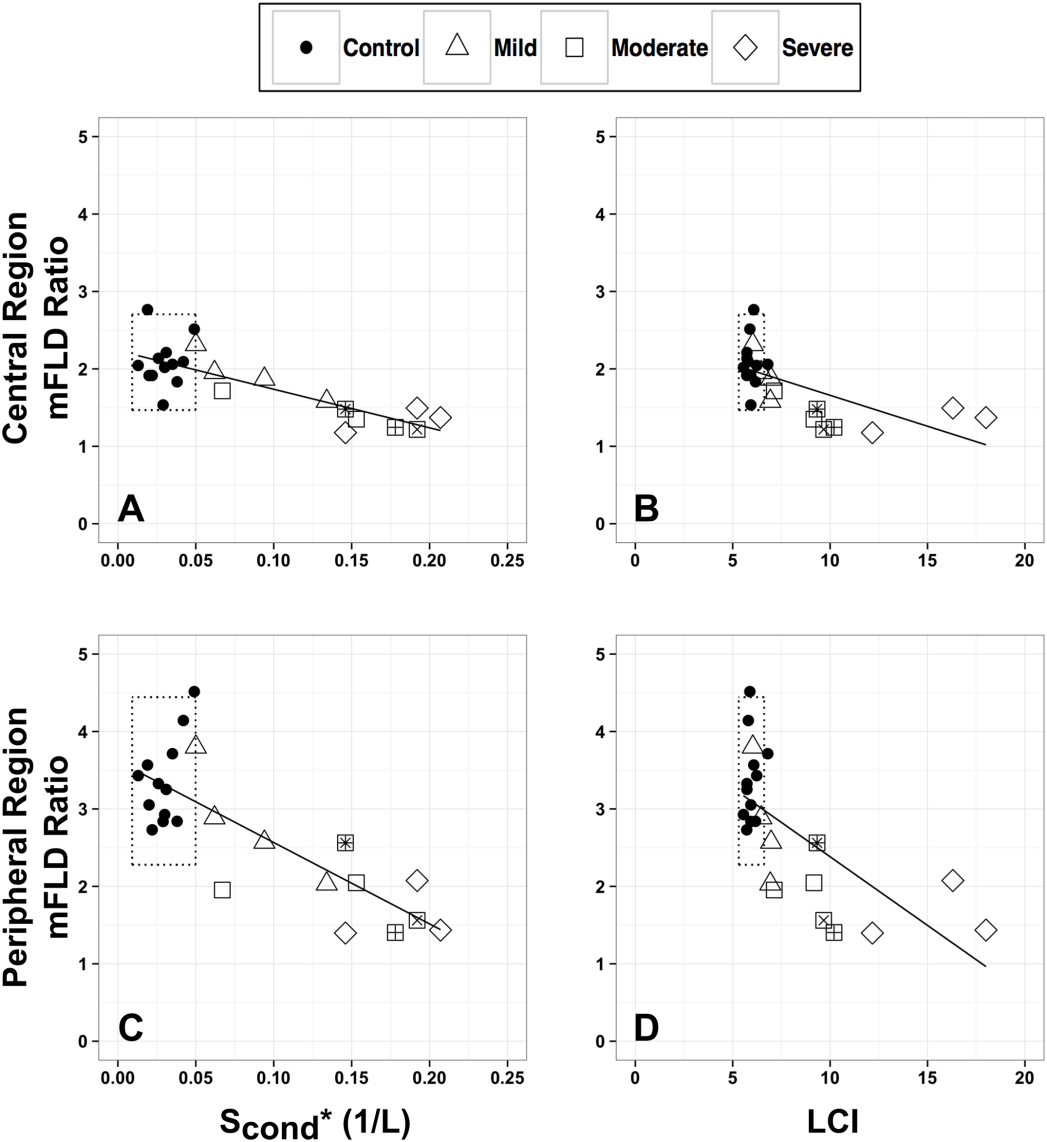
MBW indices (S_cond_*, LCI) vs. the mFLD ratio in both lung regions. (A,C) S_cond_* (1/L) and (B,D) LCI vs. mFLD ratio in the (A,B) central and (C,D) peripheral region. Data presented for 12 healthy controls 12 CF adults with stable disease (4 had mild (FEV_1_ > 80%), 5 had moderate disease (50%<FEV_1_<80%), and 3 had severe disease (FEV_1_<50%)). Dotted lines are range of control subjects in study (i.e mean ± 1.96*SD). Individual subjects with similar FEV_1_ [%pred] and LCI 2% are identified by +, * and ×.

There was a high negative correlation between the mFLD ratio (both regions) and S_cond_* for all subjects (central and peripheral: R = -0.79, p<0.001, Fig 5, panels A and C) and for CF subjects only (central: R = -0.85, p<0.001; peripheral: R = -0.75, p<0.001). The mFLD ratio in the peripheral region had a moderate negative relationship with LCI (R = -0.67, p<10^−5^, Fig 5, panel D).

Despite the small sample size (CF subjects, n = 12), a multiple regression analysis was employed to understand which combination of MRI measures might affect measurements of lung function as measured by MBW. Prior to conducting a hierarchical multiple regression, the relevant assumptions of this statistical analysis were tested. Residual and scatter plots indicated the assumptions of normality, linearity, and homoscedasticity were all satisfied. The assumption of multicollinearity was met since collinearity statistics (i.e. VIF) were all within accepted limits (threshold = 5).

A two-stage hierarchical multiple regression was conducted with S_cond_* (1/L) as the dependent variable, and a three-stage hierarchical multiple regression was conducted with LCI as the dependent variable. For S_cond_*, mFLD_TLC_ in the central region was entered in the first stage of the regression to control for the presence of excess water in the conducting airways. The mFLD ratio in the central region was entered in the second stage. Regression statistics for this model are reported in Table 5. Adding the mFLD ratio to the regression model explained an additional 31% of variance in S_cond_* and this change in R^2^ was significant, F(1,9) = 10.28, p<0.05. The most important predictor of S_cond_* was the mFLD ratio, which uniquely explained 34% of the variation in S_cond_*. The mFLD and the mFLD ratio in the central region accounted for 72% of the variance in S_cond_*.

**Table 5:** Two-stage Hierarchical Regression Analysis for Variables predicting S_cond_* for subjects with Cystic Fibrosis.

Variable	b	β	t	sr^2^	R	R^2^	ΔR^2^
**Step 1**					0.64	0.41	0.41
**Central region: mFLD_TLC_**	0.884	0.640	2.636*	0.41			
**Step 2**					0.85	0.72	0.31
**Central region: mFLD_TLC_**	-0.166	-0.120	-0.409	0.005			
**Central region: mFLD ratio**	-0.151	-0.945	-3.207*	0.342			

Note. N = 12;

* p<0.05

b (unstandardized) and β (standardized) regression coefficients, t: t-test for predictors, sr^2^: semi-partial correlation, R: correlation, R^2^: coefficients of determination, ΔR^2^: R Square Change

The mFLD ratio in the central region was entered for the first step of the regression with LCI as the dependent variable. mFLD_FRC_ in the peripheral region was entered at step 2, and the mFLD ratio in the peripheral region was entered in the final step. Regression statistics for this three-stage model are presented in Table 6. Adding the mFLD_FRC_ in the peripheral region explained an additional 25% of the variation in LCI and this change in R^2^ was also significant, F(1,9) = 5.40, p<0.05. The addition of the mFLD ratio in the peripheral region to the regression model explained an additional 31% of variance in LCI and this change in R^2^ was also significant, F(8,1) = 23.1, p<0.01. All three independent variables were significant predictors of LCI when included in the stage 3 of the regression model and accounted for 89% of the variance in LCI.

**Table 6:** Three-stage Hierarchical Regression Analysis for Variables predicting LCI for subjects with Cystic Fibrosis.

**Variable**	b	β	t	sr^2^	R	R^2^	ΔR^2^
**Step 1**					0.57	0.32	0.32
**Central region: mFLD ratio**	-6.404	-0.569	-2.187	0.32			
**Step 2**					0.76	0.58	0.25
**Central region: mFLD ratio**	-5.003	-0.444	-1.990	0.19			
**eripheral region: mFLD_FRC_**	-68.39	-0.519	-2.324*	0.25			
**Step 3**					0.94	0.89	0.31
**Central region: mFLD ratio**	14.700	1.305	3.405**	0.17			
**Peripheral region: mFLD_FRC_**	-128.982	-0.974	-6.368***	0.24			
**Peripheral region: mFLD ratio**	-9.644	-1.789	-4.806**	0.18			

Note. N = 12;

* p<0.05,

** p<0.01,

*** p<0.001

b (unstandardized) and β (standardized) regression coefficients, t: t-test for predictors, sr^2^: semi-partial correlation, R: correlation, R^2^: coefficients of determination, ΔR^2^: R Square Change

## Discussion

The main goal of this study was to combine spatial measures of lung water density with MBW measures of ventilation inhomogeneities to evaluate disease status in adults with cystic fibrosis. The study used a highly reliable and validated fast gradient echo MRI sequence to measure the regional distribution of lung water density in CF adults with stable disease and healthy controls [25, 26]. The MRI data lead to two main observations. First, mFLD_TLC_ was significantly higher in the CF group when compared to controls in both the central and peripheral region of the lung (Table 3). Since the pulmonary thoracic space at TLC (as measured by MRI) was not significantly different between groups, the additional lung water content measured in the CF group likely is the result of additional fluid within retained airway secretions, edema and infections (Fig 3). Second, mFLD_FRC_ was similar in the CF and control group, however CF patients had a significantly higher pulmonary thoracic space than controls. The higher pulmonary volume is likely due to the presence of air trapping in CF patients [30–32]. Due to the abnormal retention of air in the lung, air trapping is identified by areas of low attenuation (decreased density) when compared to surrounding tissue on expiratory CT. While the absolute amount of water present in the CF lung is higher than in the healthy lung, our MRI results will also be affected by air trapping that results in a regional reduction of lung water density at FRC. Our data might indicate that the combination of an increase in water content due to excess secretions and edema was balanced by low water content in air trapped regions, these two opposing trends resulting in no significant change in mFLD between the healthy and CF group at FRC.

The combined presence of excess water from structural abnormalities (increased density at FRC and TLC) and air trapping (decreased density at FRC) in the CF lung can be characterized by the mFLD ratio defined as the ratio between mFLD at FRC to the mFLD at TLC. Indeed, as supported by our data, such a ratio is significantly smaller in CF patients when compared with controls. This ratio may thus be a useful biomarker reflecting the heterogeneity of disease expression [33] since CF patients may exhibit one or many of the multiple structural consequences of the disease (bronchiectasis, air trapping, mucus plugging, etc) that have the potential to reduce the mFLD ratio when compared to healthy lungs.

At present, a majority of studies utilizes FEV_1_ and LCI to evaluate lung function in the CF population [16, 21, 34]. Our data showed a strong relationship between these two variables (R = -0.91, Fig 4, panel B), but also showed LCI to be elevated even in CF patients with normal FEV_1_. This is in agreement with previous studies that have shown LCI be a more sensitive measure of early lung disease than FEV_1_ both in children [35–37] and adults with CF [38]. The measure of ventilation inhomogeneities in the central airways (as measured by MBW index S_cond_*) showed an even more significant difference between these two groups (Table 2). A few studies that have evaluated ventilation heterogeneities with MBW in CF adults [16, 24, 34, 39] have indicated that the increase in ventilation heterogeneities observed in CF patients is likely due to the presence of inflammation and infection. Unlike FEV_1_, our MBW tests have shown that LCI and large-scale ventilation heterogeneities (i.e. those originating in the large and medium airways, Fig 5, panels A and C) are elevated in CF patients even for those patients with normal FEV_1_ values (>80% pred) [16, 39].

To determine the sensitivity of combined measures of regional lung structure and ventilation, we examined relationships between mFLD in the central and peripheral regions of the lung with MBW indices and FEV_1_. The strongest correlations between MBW indices and mFLD were found for MR data acquired at TLC rather than at FRC. In particular, there was a strong correlation between mFLD in the central region of the lung with S_cond_* and LCI (Fig 5, panels B and C, Table 4). These data suggest that an elevated mFLD indicates the presence of excess mucus in the conducting airways, which ultimately obstructs airflow in the large airways leading to an uneven gas distribution (e.g. elevated S_cond_*) and a longer turnover rate of alveolar gas (e.g. elevated LCI). Likewise, the mFLD ratio (both regions) had a strong correlation with S_cond_*. This highly significant correlation is present for both groups pooled together (all subjects) but also for the CF group only (Fig 5). Given that we have previously described the mFLD ratio as a quantitative metric that encompasses abnormal lung morphology in the CF lung (e.g. air trapping, retained mucus, edema, infection, and/or inflammation), results suggest that large-scale ventilation heterogeneities (S_cond_*) are not only influenced by elevated mFLD in the central region but by a conglomerate of many abnormalities (mFLD ratio) in the lung. For example, the defined central lung region contains the conducting airways and lung parenchyma. Thus, any change in the mFLD ratio of the central region will be linked to heterogeneous ventilation in the large and the small airways. Hierarchical multiple regression predicting S_cond_* has shown that by including a structural metric to the model representing the complicated nature of abnormal structure (mFLD ratio) may be a better predictor than a simple metric such as elevated lung water content in the conducting airways (Table 5).

Likewise to S_cond_*, LCI has a strong correlation with the mFLD ratio (both region) for all subjects. However, the relationship between LCI and the mFLD ratio loses significance for the CF group only (Table 4). A few studies have linked computed tomography (CT) scores of abnormal lung structure with MBW indices [38, 40, 41], where an elevated LCI was related to the presence of structural changes in the lung [42]. LCI has been considered a marker of airway infection and inflammation (small airway function) and a possible endpoint to evaluate exacerbations [43, 44]. However, recent MBW studies have shown that LCI not only reflects peripheral ventilation inhomogeneities (associated with small airways function) but also far more proximal inhomogeneities [45]. Consequently, LCI (Table 6**)** may best be predicted by multiple MRI metrics, which describe abnormal structure originating in the large and/or medium airways (central mFLD ratio), abnormal structure originating in the small airways (peripheral mFLD ratio), and peripheral mFLD at FRC (air trapping in the small airways). Due to the strong link between LCI and structural defects in the small airways, this may explain why the magnitude of LCI response to treatment in CF has been modest [39, 46–49], since a majority of treatments available to maintain disease status do not reach the small airways. Additionally, there is evidence that during an exacerbation there are regional differences in airway inflammation as detected by CT, and it is the lobes with “greatest” disease that respond to therapeutic intervention [40]. Thus, if a successful therapy is limited to specific region, a positive response might be dampened since LCI may be linked to many facets of abnormal lung structure.

Lastly, MBW metrics in combination with MRI metrics can add an additional dimension to evaluating CF patients on an individual-by-individual basis. In Fig 5, panel A, three subjects in the moderate CF group are identified by a square surrounding a unique symbol. These three subjects, with similar FEV_1_ and LCI values (“+”, “*” and “×”), have very different S_cond_* and mFLD ratios. Subjects, “+” and “×”, have ventilation and peripheral structural abnormalities that are similar to CF subjects with severe disease. Whereas, subject “*” has a lower mFLD in the central region at TLC (Fig 5, panel B) and a higher mFLD ratio in the peripheral region (Fig 5, panels C and D) which might suggest this subject has airflow obstruction originating in the large airways leading to a reduced LCI and S_cond_* when compared to CF adult with mild disease or healthy controls. Thus, given a similar therapeutic intervention, it might be possible that each of these subjects will have a unique response due to their structural and ventilation differences.

### Limitations

We acknowledge that measures of mFLD are non-specific at the current stage of development of the technique. However, it should be emphasized that even though mFLD includes abnormalities from various sources (bronchiectasis, edema, excess mucus, etc…), our preliminary data clearly show that the measure of FLD can differentiate between a healthy lung and a CF lung and this measure can be linked with disease status. Furthermore, commonly used clinical parameters including spirometric pulmonary function testing have limited sensitivity and provide no regional information.

The mGRE imaging sequence used in this study has been validated in a previous study by our group) [26], and provides a measure of total lung water content that includes water from all sources including blood and tissue. Thus, we are unable to distinguish between intravascular and extravascular lung water. However, we do not anticipate that pulmonary vascular dysfunction (pulmonary hypertension) will have a large effect on the mFLD measures and their link with ventilation heterogeneity. In a previous study by our group, we used a model in which 20mL/kg of saline was rapidly infused intravenously in healthy control subjects to replicate pulmonary interstitial edema which increases pulmonary arterial pressure in humans [50]. They found that the infusion of saline increased the total thoracic fluid content by 13% and increased perfusion in the nondependent lung by 16%. If 13% of the measured mFLD were hypothetically entirely assigned to intravascular water, then mFLD_FRC_ and mFLD_TLC_ would be reduced in the CF group by 0.02 and 0.01, respectively. Thus, it is unlikely that an intravascular water component of mFLD will have a significant impact on mFLD, the mFLD ratio, and on the relationship with MBW indices since.

With our current imaging voxel size (35 mm^3^), we can evaluate water content in one-fifth to one-half of an acinus (~200 mm^3^) depending on lung volume. So, it is unlikely that we can accurately detect changes in the very peripheral airways. This is likely why our results show very weak to moderate relationships between our MRI measures and S_acin_*.

The pulmonary thoracic space, lung gas and tissue volumes, was determined via manual segmentation of MR images. Due to the large imaging slice thickness, some regions of the lung were excluded due to partial volume effects where some regions in the image contained both signal from chest wall and/or heart along with lung signal. Thus, lung tissue anterior and posterior to the heart and lung tissue in the most lateral imaging slices were excluded from the analysis. The amount of the tissue excluded varied with the size of the subject is more of an issue at FRC. As a result, pulmonary thoracic space measures do not correlate with lung volume as measured by MBW. Regardless, our MRI measures of lung water density do reflect regional lung structure since ROIs contain a majority (>75%) of lung tissue.

The MBW data have been collected with a custom-built system similar to that used in other research laboratory with which a significant number of data have been collected over the years that will serve as basis for comparison [12–15, 19, 20, 24, 39]. However, we acknowledge the need for switching to commercially available MBW equipment that could be used in any clinical research unit.

The MBW tests were performed with the subject sitting while the MR imaging was done with the subject in the supine position. Thus, there is a potential for a body position effect between the two measures. We performed the MBW tests in the seated posture to follow the protocol currently used in the CF community [21, 22, 42]. The MRI measurements that aimed at quantifying the amount of excess fluid in the lungs of CF patients (and not ventilation which would be affected by posture) were performed at predetermined static lung volumes. It is unlikely that the spatial distribution of excess fluid was significantly altered during the relatively short period of imaging (<40 minutes) which is demonstrated by the test-retest reliability measures (R^2^ = 0.9) and thus it is unlikely that the different postures significantly altered the correlation between MRI data and MBW indices.

## Conclusion

In summary, the combined technique of evaluating MBW (ventilation) and MRI (structural abnormalities) metrics may allow us to characterize a subject’s disease status and evaluate regions that are difficult to assess (peripheral region) with traditional lung function values alone (FEV_1_ and LCI). With the advances in imaging in general and the ability to characterize and quantify CF in greater detail, imaging will likely play an increasing role in the improved understanding of the disease process and will serve as a biomarker for the development of new treatments. Finally, because of the steady increase in the median survival of CF patients well into adulthood, it is becoming important to develop radiation-free diagnostic options that can be used in the longitudinal monitoring of the disease. This study demonstrates the potential for future additional development into the diagnostic potential of combining “global” or whole lung indices as measured by MBW and “regional” MRI measures of abnormal lung morphology (central, peripheral, lobular), which could be implemented in the clinic.

## Supporting Information

S1 File Participant-level spirometry, multiple breath washout (MBW), and fractional lung density (FLD) data. (PDF)
