# Supplementary material for: Characterizing Lung Disease in Cystic Fibrosis with Magnetic Resonance Imaging and Airway Physiology
Source: PLoS One. 2016 Jun 23;11(6):e0157177. doi: 10.1371/journal.pone.0157177 (PMC4919047; doi:10.1371/journal.pone.0157177)
Supplement: S1 File — (PDF) [file pone.0157177.s001.pdf]

	GENERAL DATA					PFTs				MBW DATA			MRI DATA					
	Subject ID	age	gender	height (cm)	weight (kg)	FEV1 (%pred)	FVC (%pred)	FEV1/FVC (%pred)	FEF25-75 (%pred)	Scond* (1/L)	Sacin* (1/L)	LCI	Central: mFLD at FRC	Peripheral: mFLD at FRC	Central: mFLD at TLC	Peripheral: mFLD at TLC	Central: mFLD Ratio	Peripheral: mFLD Ratio
CF	**1**	24	M	183	76	32	44	71	10	0.207	0.602	18.00	0.27	0.17	0.20	0.12	1.37	1.43
CF	**2**	26	M	165	61	46	69	67	19	0.192	0.964	16.30	0.20	0.12	0.14	0.06	1.49	2.08
CF	**3**	39	F	173	56	48	65	73	22	0.146	0.665	12.17	0.22	0.17	0.19	*0.12*	1.18	1.40
CF	**4**	27	F	170	59	53	66	80	23	0.178	0.279	10.20	0.27	0.19	0.21	0.14	1.24	1.40
CF	**5**	24	M	168	62	57	66	86	32	0.192	0.122	9.67	0.29	0.20	0.24	*0.13*	1.22	1.56
CF	**6**	32	M	177	68	58	86	67	22	0.146	0.257	9.33	0.20	0.13	0.14	0.05	1.48	2.56
CF	**7**	23	F	175	64	73	88	82	45	0.153	0.179	9.17	0.24	0.18	0.17	0.09	1.35	2.05
CF	**8**	29	F	154.9	61.7	80	81	100	80	0.067	0.145	7.13	0.29	0.23	0.17	0.12	1.72	1.95
CF	**9**	30	F	165	56	86	84	102	103	0.134	0.091	6.93	0.25	0.20	0.16	*0.10*	1.58	2.03
CF	**10**	37	F	162.5	63.5	99	108	91	79	0.062	0.093	6.47	0.28	0.19	0.14	0.07	1.95	2.89
CF	**11**	27	F	163	49	99	101	99	110	0.094	0.152	6.97	0.23	0.20	0.12	0.08	1.87	2.57
CF	**12**	23	M	182.9	76	104	103	100	97	0.050	0.136	6.03	0.24	0.18	0.10	0.05	2.31	3.80
healthy	1	29	M	178	79	87	88	99	77	0.020	0.104	5.93	0.22	0.18	0.12	0.06	1.91	3.05
healthy	2	41	M	175	81	87	94	92	65	0.035	0.199	6.80	0.23	0.17	0.11	0.05	2.06	3.71
healthy	3	23	M	175	64	93	104	90	72	0.013	0.124	6.23	0.21	0.15	0.10	0.04	2.04	3.43
healthy	4	44	F	164	61	105	107	98	94	0.038	0.110	6.17	0.20	0.17	0.11	0.06	1.83	2.84
healthy	5	23	F	165	59	98	96	103	102	0.022	0.089	5.72	0.18	0.16	0.09	0.06	1.91	2.73
healthy	6	27	M	185.4	81.6	93	88	105	95	0.029	0.113	5.93	0.21	0.17	0.14	0.06	1.53	2.84
healthy	7	23	M	172	64	96	90	106	106	0.026	0.112	5.73	0.21	0.18	0.10	0.06	2.14	3.33
healthy	8	42	F	163	79.6	102	102	100	108	0.031	0.090	5.73	0.25	0.21	0.11	0.06	2.21	3.25
healthy	9	37	M	177	90	86	86	100	88	0.042	0.069	5.80	0.26	0.22	0.12	0.05	2.09	4.14
healthy	10	42	F	160	71	87	98	88	58	0.049	0.145	5.88	0.27	0.22	0.11	0.05	2.51	4.52
healthy	11	29	F	178	63.5	92	86	105	121	0.019	0.096	6.08	0.30	0.25	0.11	0.07	2.76	3.57
healthy	12	30	F	173	64	93	94	98	94	0.030	0.053	5.55	0.22	0.20	0.11	0.07	2.02	2.93
